# Targeting skin barrier repair: mechanisms of action, therapeutic evidence, and clinical translation challenges of mesenchymal stem cell-derived exosomes

**DOI:** 10.1186/s13287-026-04941-6

**Published:** 2026-05-20

**Authors:** Yujin Li, Jian Huang, Zhibing Fu, Lihua Gao, Xiaoliang Tong, Lu Zhou, Jinrong Zeng, Lina Tan

**Affiliations:** Central South University Third Xiangya Hospital, Central South University, Chang Sha, China

**Keywords:** Mesenchymal stem cell-derived exosomes, Skin barrier repair, Cell-free therapy, Skin homeostasis, Clinical translation

## Abstract

Dysfunction of the skin barrier is a central pathological feature in dermatology, driving the need for innovative repair strategies. Mesenchymal stem cell-derived exosomes (MSC-exos) represent a promising cell-free therapeutic paradigm, leveraging their innate cargo to modulate regeneration and immune responses. This review systematically examines the multifaceted role of MSC-exos in restoring skin barrier integrity. We delineate their molecular mechanisms in repairing physical, immunological, and microbial barrier components, supported by evidence from preclinical disease models. The influence of MSC source and preconditioning on exosome efficacy is analyzed, alongside emerging bioengineering approaches. Crucially, we identify and discuss the key translational challenges—including standardization, scalable manufacturing, and regulatory pathways—that must be addressed to advance these nanotherapeutics toward clinical application. This synthesis provides a critical framework for future research aimed at harnessing MSC-exos for targeted barrier repair.

**Graphical abstract:**

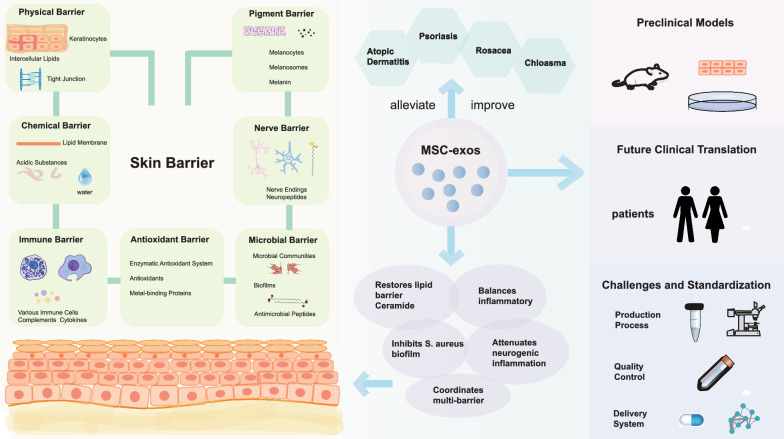

## Introduction

Mesenchymal stem cell (MSCs) are characterized by their capacity for self-renewal and multi-lineage differentiation. They can be isolated from various tissues, with bone marrow (BMSCs), adipose tissue (ASCs), and umbilical cord (UCMSCs) being the most extensively studied sources. Among these, umbilical cord– and placental–derived MSCs exhibit higher proliferative potential and are associated with fewer ethical concerns [1]. MSCs possess well-documented anti-inflammatory and immunomodulatory properties, coupled with low immunogenicity [2, 3]. Furthermore, their expression of various chemokine receptors enables targeted migration and homing to sites of tissue injury, where they participate in repair processes [4]. It is now established that the therapeutic effects of MSCs are mediated primarily through paracrine signaling, with exosomes serving as key effector vehicles of this action [5].

Exosomes are small, extracellular vesicles with a diameter of approximately 30–150 nm, released upon the fusion of multivesicular bodies with the plasma membrane [6]. They serve as pivotal mediators of intercellular communication, facilitating the horizontal transfer of bioactive molecules—including proteins, lipids, and nucleic acids (such as mRNA and miRNA)—between cells [6, 7]. This cargo is not randomly packaged; its loading into intraluminal vesicles within multivesicular bodies is a regulated process, primarily orchestrated by the Endosomal Sorting Complex Required for Transport (ESCRT) machinery [8]. The ESCRT complexes (ESCRT-0, -I, -II, -III) and associated proteins like Vps4 and Alix work sequentially to recognize ubiquitinated cargos, drive membrane invagination, and mediate vesicle scission [8]. Following their release, exosomes are characterized by a conserved set of membrane tetraspanins (e.g., CD9, CD63, CD81) and cytosolic proteins (e.g., TSG101, Alix), which are commonly used as identification markers [9].

Studies indicate that MSC-exos exhibit therapeutic efficacy comparable to, or even surpassing, that of their parent cells in various disease models [10]. Both MSC-exos and MSCs can adapt to specific microenvironments, eliciting biological responses that restore and maintain homeostasis. Their regulatory impact appears proportional to the degree of homeostatic imbalance, enabling precise modulation without overcorrection and effectively normalizing pathological states [11]. This suggests a wide therapeutic safety window for MSC-exos. Furthermore, as nanoscale vesicles, MSC-exos offer practical advantages in transport, storage stability, and the ability to traverse capillary networks via systemic circulation to reach target tissues. Compared with whole MSCs, MSC-exos allow for more quantifiable dosing and are associated with significantly lower risks of embolism, tumorigenicity, and chromosomal abnormalities, presenting a safer therapeutic profile [12].

### Search strategy and selection criteria

A comprehensive literature search was conducted using the Web of Science Core Collection and PubMed databases (January 2000–December 2023). To capture relevant studies, core search terms included: “mesenchymal stem cells” OR “MSC”, “exosomes” OR “extracellular vesicles”, and “skin barrier” OR “repair”. Given the evolving nature of direct research on MSC-exos and skin barrier repair, the search was expanded to encompass key related pathological processes: skin microbiota, inflammation, oxidative stress, and skin metabolism. Medical Subject Headings (MeSH) terms were employed to enhance precision.

The selection focused on peer-reviewed systematic reviews, meta-analyses, and original research articles. Priority was given to studies that investigated MSC-exos’ modulation of the aforementioned processes and demonstrated a link to the restoration of physical or immune barrier function, particularly within the context of atopic dermatitis, psoriasis, rosacea, and melasma. Screening proceeded in two phases: initial title/abstract review followed by full-text assessment. Articles were excluded if they were not peer-reviewed, focused on irrelevant exosome sources, or employed models with low translational relevance. Manual screening of reference lists from key articles identified additional pertinent studies.

### Skin diseases characterized by barrier dysfunction

#### The multifaceted skin barrier: a dynamic network central to dermatological health (Fig. 1)

**Fig. 1:**
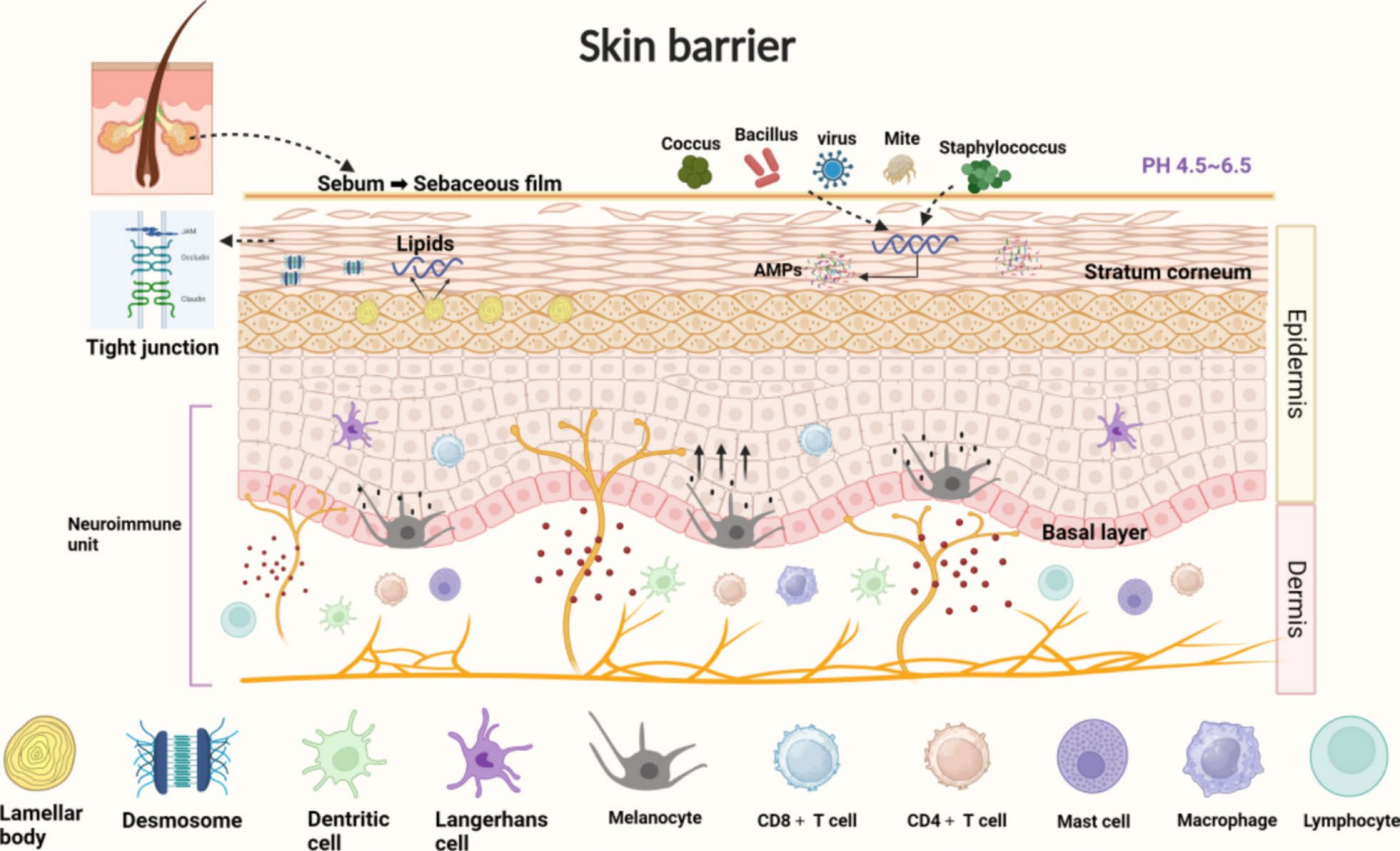
Composition of the skin barrier

The skin, the body's largest organ, serves as the primary interface with the external environment, providing essential protection against physical, chemical, and biological threats. This critical protective role is fulfilled by the skin barrier, which is best understood not as a single entity but as a dynamic and integrated network of interdependent components. The contemporary view has evolved from a narrow focus on the physical barrier—chiefly the stratum corneum and its lipid matrix—to encompass the immune, chemical, microbial, neural, and pigmentary dimensions that constitute a fully functional barrier system (Fig. 1) [13]. These components operate not in isolation but through constant, synergistic crosstalk to maintain cutaneous homeostasis [14].

The integrity of this multidimensional network is fundamental to skin health. Perturbations within one component can initiate dysfunction in others, establishing a self-perpetuating cycle of damage and inflammation. Consequently, barrier dysfunction is a central pathogenic driver in a spectrum of dermatoses, including atopic dermatitis, psoriasis, acne, and melasma [15, 16]. This underscores the therapeutic imperative for strategies that aim to holistically restore barrier integrity, addressing the interconnected network rather than an isolated dimension, for effective disease intervention and prevention.

The skin barrier is a multifaceted and integrated system composed of several functionally distinct yet interdependent components. The physical barrier is primarily constituted by the stratum corneum, sebaceous film, intercellular lipids, and specialized junctional complexes (tight junctions and desmosomes). The chemical barrier is formed through lipids and acidic metabolites derived from both skin secretions and resident microbiota. The pigment barrier involves melanin, which is transferred to keratinocytes and shed during their normal differentiation and desquamation process. The microbial barrier encompasses the diverse community of commensal bacteria, viruses, and mites that colonize the skin surface. Finally, the neuroimmune barrier represents a highly integrated network where sensory nerve fibers and immune cells interact closely to coordinate defensive responses. These components work in concert to maintain the integrity, homeostasis, and overall functionality of healthy skin.

#### Atopic dermatitis

Atopic dermatitis (AD) is a chronic, relapsing inflammatory skin disease characterized by eczematous lesions and severe pruritus. Its pathogenesis involves a complex triad: genetic predisposition, epidermal barrier dysfunction, and immune dysregulation. Lesion morphology and distribution vary with age, evolving from acute, exudative patches in infants to chronic, lichenified plaques in adults. Critically, epidermal barrier impairment is now recognized as a primary event that facilitates allergen penetration and subsequent immune activation [17].

A substantial subset of AD patients carries loss-of-function mutations in genes encoding key epidermal structural proteins, such as filaggrin (FLG), keratins, and loricrin. These mutations compromise the physical barrier, leading to increased transepidermal water loss (TEWL) and enhanced permeation of allergens and irritants, which trigger and sustain inflammation [18]. This defective barrier also promotes skin dysbiosis, typified by Staphylococcus aureus over-colonization, loss of microbial diversity, and reduced antimicrobial peptide (AMP) production, further disrupting immune homeostasis [19, 20].

Stratum corneum impairment in AD is mechanistically linked to profound alterations in ceramide composition. Intercellular lipids, essential for barrier integrity, consist of approximately 50% ceramides. In AD, not only is total ceramide content reduced, but the molecular profile is also aberrant: levels of short-chain ceramides (carbon chain length, C < 42) are elevated, while long-chain ceramides (C > 42) are deficient in both lesional and non-lesional skin [19]. Th2 cytokines, particularly IL-13, can directly inhibit ceramide chain elongation, creating a direct link between immune activation and lipid barrier defects [21]. Notably, the pro-apoptotic short-chain ceramide C16 is elevated, reflecting impaired keratinocyte terminal differentiation and contributing to inflammation and xerosis [22]. Correcting these ceramide abnormalities represents a fundamental therapeutic strategy to restore barrier function and mitigate the need for chronic anti-inflammatory therapies [23].

FLG deficiency, primarily due to common genetic mutations, is a major heritable risk factor for AD [24]. It disrupts epidermal integrity and the physiological crosstalk between skin barrier components, creating a pro-inflammatory milieu that facilitates allergen penetration and immune sensitization [25]. Upon barrier breach, allergens trigger keratinocytes to produce thymic stromal lymphopoietin (TSLP), which potently exacerbates Th2 responses in a vicious cycle. FLG expression itself is suppressed by Th2 cytokines, creating a feedback loop [26]. Furthermore, FLG degradation products (natural moisturizing factors) are vital for stratum corneum hydration, acidification, UV defense, and suppression of pro-inflammatory cytokines, underscoring its multifaceted role in barrier maintenance [27].

AD is a quintessential cytokine-driven disease, featuring elevated levels of Th2 cytokines (e.g., IL-4, IL-13, IL-31). TSLP is a pivotal cytokine linking barrier defects to Th2 polarization and pruritus [28]. This immune dysregulation directly exacerbates barrier failure. Th2 cytokines suppress keratinocyte production of AMPs, thereby facilitating S. aureus colonization, which in turn secretes superantigens and proteases that further amplify immune activation and barrier damage [29, 30]. The bacterial burden of S. aureus often correlates directly with clinical disease severity [29].

In summary, AD pathogenesis is driven by a self-perpetuating cycle wherein genetic and acquired barrier defects lead to immune hyperactivation (predominantly Th2), which subsequently further damages the barrier and alters the skin microbiome. This refined understanding directly informs modern therapeutic strategies, including biologics that target the IL-4/IL-13 axis and emerging therapies aimed at upstream pathways [31].

#### Psoriasis

Psoriasis, most commonly presenting as plaque psoriasis, is a chronic, immune-mediated dermatosis driven by genetic susceptibility and environmental factors. It is clinically defined by well-demarcated, scaly, erythematous plaques that may be localized or widespread, often exacerbating in winter. The pathogenesis involves a self-perpetuating cycle between a compromised physical skin barrier and sustained immune hyperactivation. Initial defects in barrier components—such as reduced tight junction (TJ) protein expression and dysregulated ceramide metabolism—predispose keratinocytes to exaggerated responsiveness. These keratinocytes are subsequently driven into a state of hyperproliferation and aberrant differentiation by autocrine and paracrine signals from activated immune cells, sustaining a pro-inflammatory microenvironment [32]. This process is underpinned by a profound immune dysregulation, characterized by enhanced pro-inflammatory activation alongside impaired anti-inflammatory suppression [33].

The immunopathology is hallmarked by dysregulated T‑cell activity, with central roles for CD8+ T cells and T‑helper 17 (Th17) cells. The release of key cytokines, particularly IL‑17 and IL‑23, directly perturbs keratinocyte differentiation, promotes hyperproliferation, and amplifies inflammation. Keratinocytes, in turn, act as potent amplifiers by producing a cascade of pro‑inflammatory mediators—including IL‑6, IL‑8, IL‑25, IL‑36, and tumor necrosis factor‑alpha (TNF‑α)—that recruit and activate additional immune cells, thereby reinforcing positive feedback loops that intensify the inflammatory cascade [34]. Histologically, lesional skin shows elevated expression of proliferation markers (e.g., Ki‑67) and dysregulation of keratinocyte differentiation markers. Notably, TJ dysfunction is evident even in early disease, suggesting barrier impairment may contribute to disease initiation [33].

Biologic therapies targeting the IL‑17 and IL‑23 pathways have revolutionized treatment, demonstrating high efficacy in clearing lesions. However, their use can be limited by class-specific adverse effects. These include an increased risk of infections (e.g., from neutropenia), contraindications in patients with certain comorbid conditions like inflammatory bowel disease (particularly for IL‑17 inhibitors), and potential over‑suppression of protective immune responses [35]. Consequently, the exploration of next‑generation immunomodulators, such as MSC‑exos, either as monotherapy or in rational combination with existing biologics, represents a promising strategy to maintain efficacy while potentially mitigating adverse effect profiles.

#### Rosacea

Rosacea is a chronic, relapsing inflammatory dermatosis primarily affecting the facial vasculature and pilosebaceous units. Its clinical presentation encompasses a spectrum of features, including persistent centrofacial erythema, flushing, inflammatory papules and pustules, telangiectasia, and, in severe cases, phymatous changes such as rhinophyma. Pathogenetically, dysregulation of the neuro‑immune‑vascular interface is considered a central initiating event. Neurovascular dysfunction and sebaceous gland abnormalities contribute to increased TEWL, elevated skin surface pH, and heightened susceptibility to exogenous triggers. Neurogenic inflammation is pivotal in driving the characteristic chronic and recurrent disease course [36]. Molecular studies confirm significant epidermal barrier impairment in patients with rosacea compared to healthy controls [37].

Mast cells, which form functional neurovascular units with sensory nerves and blood vessels, are increased in density across all rosacea subtypes. Morphometric and transcriptomic analyses indicate robust nerve‑mast cell interactions, with lesional skin showing upregulation of the histamine receptor HRH2—a key mediator of vasodilation [38]. Beyond neuroimmune dysregulation, alterations in the cutaneous microbiota are implicated. While the role of Cutibacterium acnes remains to be fully clarified, Staphylococcus epidermidis strains isolated from rosacea patients demonstrate distinct pathogenic potential, secreting more proteins and exhibiting more consistent β‑hemolytic activity compared to strains from healthy individuals [39].

At the molecular level, rosacea-affected skin exhibits increased activation of transient receptor potential vanilloid channels (TRPV1, TRPV4) and elevated expression of key mediators. These include the antimicrobial peptide LL‑37, kallikrein‑5 (KLK5), TNF‑α, vascular endothelial growth factor (VEGF), and interleukin‑1 alpha (IL‑1α), which collectively drive neurogenic inflammation, vasodilation, and altered pain perception [40]. Furthermore, signal transducer and activator of transcription 3 (STAT3) is highly expressed in both patients and experimental models of barrier damage. STAT3 promotes immune cell infiltration by regulating cytokine and chemokine networks, serving as a critical molecular link between barrier dysfunction and sustained immune dysregulation [41].

While topical and systemic therapies remain cornerstone treatments for inflammatory lesions, and light‑based devices can address associated telangiectasia and dyspigmentation, there remains a clear need for safer, more effective long-term strategies [42]. Given the intertwined pathology of barrier impairment and neuro‑immune‑vascular activation, therapeutic approaches capable of concurrently promoting barrier repair and modulating this dysregulated axis hold significant promise for improving outcomes in rosacea.

#### Chloasma

Chloasma, commonly known as melasma, is an acquired hyperpigmentation disorder characterized by symmetric, brownish patches on sun-exposed facial areas, with a pronounced predominance in women. Its pathogenesis is multifactorial, involving a complex interplay of ultraviolet (UV) radiation, hormonal influences, and genetic susceptibility.

Histopathological examination reveals significant dermal alterations, including an increased mast cell count and activation of various immune factors. Degradation of the extracellular matrix compromises the basement membrane, facilitating the migration of melanin and melanocytes into the dermis—a process implicated in the condition's refractory and recurrent nature [43]. Consequently, therapeutic strategies that target basement membrane integrity and restore immune homeostasis are considered crucial. Biophysical assessments indicate that lesional skin exhibits a thinner stratum corneum and impaired barrier recovery. Paradoxically, there is an upregulation of genes related to barrier function, highlighting a complex and non-linear association between melasma and skin barrier dysfunction [44].

UV radiation serves as the primary exogenous trigger and exacerbating factor. Exposure upregulates VEGF and stem cell factor (SCF), which promote both angiogenesis and melanogenesis; a positive correlation exists between microvascular density and the severity of hyperpigmentation [44]. Chronic UV exposure also depletes epidermal free fatty acids and triglycerides, thereby impairing the lipid barrier [44]. Furthermore, UV radiation increases the expression of the POMC and EDN1 genes within sebaceous gland cells. These genes encode precursors for pro‑pigmentary factors such as α‑melanocyte‑stimulating hormone (α‑MSH), directly implicating this pathway in UV‑induced pathogenesis [45].

Evidence supports the presence of a low-grade inflammatory state in melasma, with lesional skin showing elevated levels of FLG and IL‑17. This suggests a link between inflammatory processes and the disruption of normal melanin turnover and retention [46]. Notably, the ceramide profile is also altered: levels of very‑long‑chain and ultra‑long‑chain ceramides are significantly increased in affected skin, while specific lipid species are reduced in areas of high melanocytic activity. These lipidomic shifts may actively contribute to the dysregulated microenvironment that promotes hyperpigmentation progression [47]. Collectively, these histopathological, vascular, inflammatory, and lipid abnormalities converge to create a self-sustaining cycle that underlies the persistence of melasma.

### Mesenchymal stem cell-derived exosomes in skin barrier repair (Fig. 2., Table 1)

**Fig. 2:**
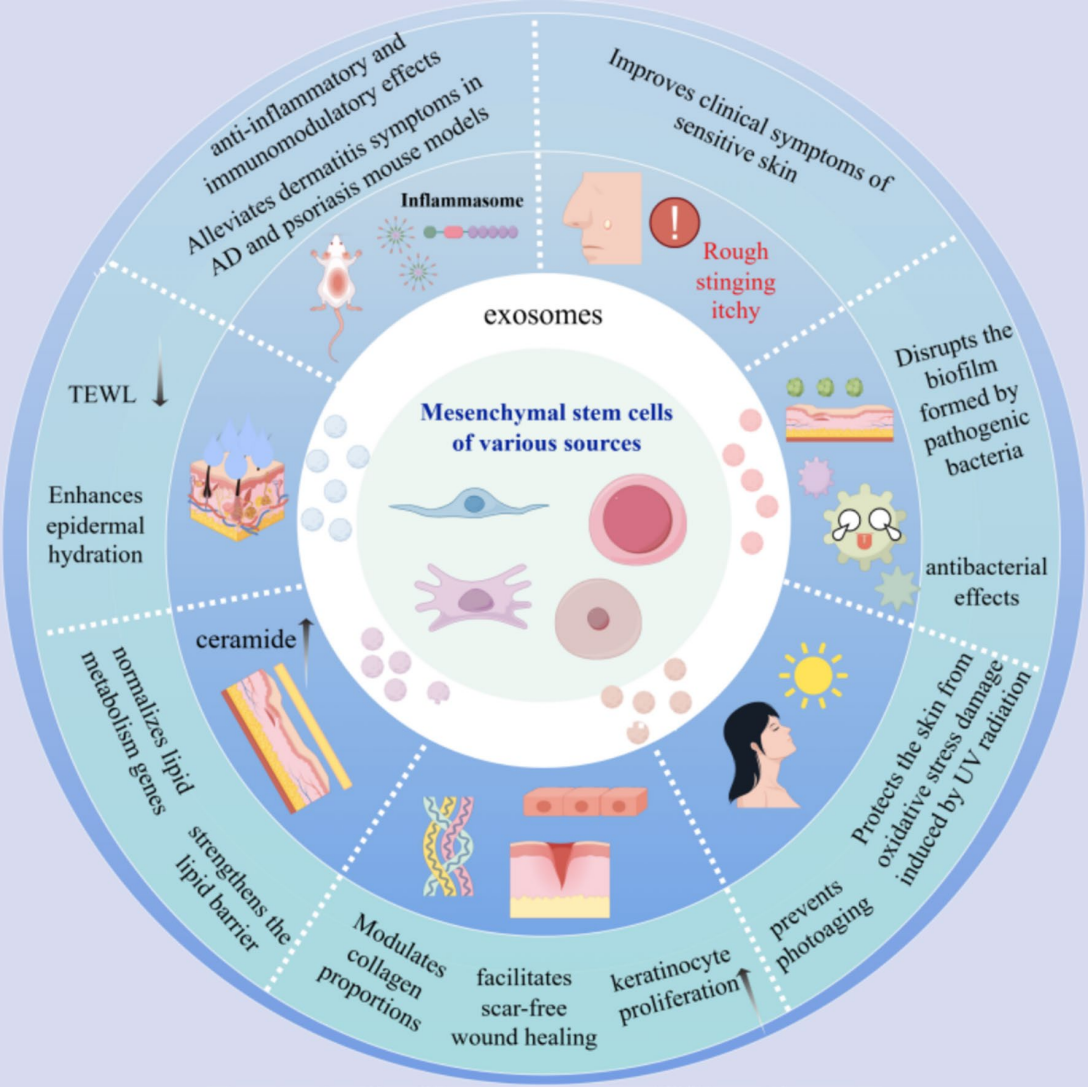
Primary Functions of Exosomes Derived from Various Sources of Mesenchymal Stem Cells in Skin-Related Research. 1. Enhances epidermal hydration and reduces TEWL. 2.Induces ceramide synthesis, normalizes lipid metabolism genes, and strengthens the lipid barrier. 3.Modulates collagen proportions, promotes keratinocyte proliferation and synthesis of key skin proteins, and facilitates scar-free wound healing. 4.Protects the skin from oxidative stress damage induced by UV radiation, maintains epidermal pigment homeostasis, and prevents photoaging. 5.Disrupts the biofilm formed by pathogenic bacteria on the epidermis, promotes the secretion of AMPs, and exerts antibacterial effects. 6.Improves clinical symptoms of sensitive skin (e.g., roughness, stinging, itching). 7.Alleviates dermatitis symptoms in AD and psoriasis mouse models, demonstrating anti-inflammatory and immunomodulatory effects

**Table 1:** Summary of evidence on mesenchymal stem cell exosomes for repairing skin barrier functions

Skin Barrier	Exosome Source	Key Findings	Study Model & Design	Evidence tier	Implications	Limitations
Physical barrier	hASCs	Repairs barrier: ↓TEWL, ↑hydration, ↑ceramide synthesis [48]	SKH-1 mouse chronic oxazolone AD; RCT, multi-dose + control	**High** (Preclinical)	Novel dual “barrier-immune” repair strategy	Model simplifies human AD; s.c. route unproven; active components unknown
		Heals wounds: ↑Type III collagen ratio (↓scar width); additive benefit of topical + IV delivery [49]	ICR mouse, full-thickness wound; RCT	**Medium–High** (Preclinical)	Validates topical therapy; combined strategy shows synergy	Mouse scarring mechanism differs from humans (e.g., hypertrophic scars)
	hAECs	Enhances repair: ↑fibroblast migration/proliferation, modulates ECM, ↑orderly collagen alignment [50]	SD rat full-thickness wound; human dermal fibroblasts (in vitro)	**Medium–High** (Preclinical)	New candidate for scar-minimizing therapy	“Scarless healing” is model-specific, not directly translatable
	iPSC-iMSCs	Boosts keratinocytes: iMSC-exos > MSC-exos via ERK1/2; s.c. exosomes internalized in vivo [51]	In vitro: HaCaT & fibroblasts; In vivo: s.c. tracer (mice)	**Medium** (Preclinical)	iPSCs as a scalable, standardized exosome source	Lacks functional wound model; caution extrapolating cell-line data
Pigmented barrier	hAMSC	Lightens pigmentation: Inhibits melanogenesis (α-MSH/UVB) & promotes melanosome degradation via autophagy; key effector miRNAs: -181a-5p, -199a [52]	Models: B16F10 cells; mouse ear UVB model; 3D human skin substitute	**Medium–High** (Preclinical)	Dual-pathway (“block synthesis + clear”) strategy with defined molecular targets (miRNAs)	Exosome-only efficacy data needed; mouse model differs from complex human conditions (e.g., melasma)
Microbial barrier	eMSCs	Fights infection: Equine MSC-CM directly disrupts MRSA biofilm; CCL2 within CM ↑ keratinocyte AMPs to enhance innate immunity [53]	Ex vivo: Equine skin explant (MRSA biofilm); In vitro: primary equine keratinocytes	**Medium** (Preclinical)	Novel “direct + host-directed” strategy against drug-resistant wound infections	Species-specific (equine); active components are in CM, not purified exosomes. microenvironment; the effective substance is a CM, not purified exosomes
Nerve barrier	hUC-MSCs	Relieves sensitive skin: Improves objective symptoms & repairs epidermal barrier; safe in 28-day trial (no SAEs) [54]	**Human trial: Single-arm, self-controlled pilot study**	**High (Clinical)**	Direct proof of safety & efficacy for topical use in humans	**Small, homogeneous cohort; short follow-up; mechanism of action unclear**
Immune barrier	hASCs	Combats AD: ↓clinical score, ↓serum IgE/eosinophils, ↓skin inflammation (multiple cytokines) [55]	Model: NC/Nga mice, house dust mite-induced AD; Design: Multi-dose, IV/SC	**High** (Preclinical)	Validates efficacy in a mainstream model; demonstrates multi-target (Th2/IL-23/IL-31) potential	Donor variability; long-term safety unknown
	hT-MSCs	Tames mast cells: Inhibits activation (TLR7-mediated) & skin inflammation; exosomal miRNAs target M-CSF/IL-8 [56]	Models: TLR7-stimulated human mast cell line; mouse imiquimod model (s.c.)	**Medium–High** (Preclinical)	Novel rationale for TLR7-driven disorders (e.g., psoriasis subtypes, pruritus)	miRNA-phenotype link is correlative; lacks causal functional validation
	hUC-MSCs	Ameliorates psoriasis: Improves phenotype, inhibits IL-23/IL-17 axis & dendritic cell activation [57]	**Model: Imiquimod-induced mouse dermatitis; Validation: HaCaT & dendritic cells in vitro**	**Medium–High (Preclinical)**	Novel cell-free strategy targeting the core therapeutic axis of psoriasis	Acute chemical model differs fundamentally from chronic human disease
	cASCs	Works across species: Canine MSC-exos repair barrier & modulate itch signaling in a mouse AD model [58]	Model: DNCB-induced mouse AD, for cross-species assessment	**Medium–High** (Preclinical)	A promising cell-free candidate for veterinary use (canine AD)	Preclinical study aimed at veterinary translation
	eMSCs	Induces immune tolerance: Mediates antigen-dependent suppression of GVHD & improves survival [59]	Models: Mouse CD4 + T cells in vitro; humanized mouse GVHD model in vivo	**High** (Preclinical)	Reveals critical prerequisite (antigen presentation) for precise immunotherapy	GVHD model differs immunologically from common skin diseases
Antioxidant barrier	hUC-MSCs	Shields from photo-damage: Activates NRF2 pathway to resist oxidative stress & boost antioxidant capacity [60]	**Model: UV-induced damage in wild-type vs. Nrf2-knockout mice**	**High (Preclinical)**	Strategy to combat photoaging by boosting endogenous NRF2 defense	Acute model ≠ chronic photoaging; vs. conventional antioxidants (e.g., Vit C/E) unknown; active component unclear

To clearly present the evidence strength and translational potential of the included studies, the key mechanistic research discussed in the text is summarized in this table. It details the study models, evidence levels, and major limitations for clinical translation, aiming to help readers critically assess the findings. The evidence levels were rated with reference to the GRADE framework, integrating the rigor of study design, clinical relevance of the models, and depth of mechanistic exploration: High = rigorous animal/clinical study; Medium–High = systematic animal study; Medium = in vitro or preliminary animal study [61, 62]

Human Adipose-derived Stem Cells (hASCs);Human Amniotic Epithelial Stem Cells (hAECs)

Induced Pluripotent Stem Cell-derived MSCs (iMSCs);Human Tonsil-derived MSCs (hT-MSCs)

Human Umbilical Cord MSCs (hucMSCs);Canine Adipose-derived Stem Cells (cASCs);Equine MSCs (eMSCs)

#### Restoring the physical and chemical barrier

The integrity of the skin's primary defense relies on the synergistic function of the physical barrier—comprising the stratum corneum and tight junctions—and the chemical acid mantle. MSC-exos facilitate the repair of this integrated structure through multifaceted mechanisms that target lipid metabolism, structural protein expression, and cellular regeneration.

Compelling evidence indicates that MSC-exos directly rectify core lipid defects. In a murine model of oxazolone-induced atopic dermatitis, subcutaneous injection of adipose-derived MSC-exos (ASC-exos) significantly reduced TEWL and increased stratum corneum hydration. Mechanistically, treatment promoted the de novo synthesis of ceramides and dihydroceramides. This biochemical finding was corroborated by ultrastructural observations revealing an increased number of lamellar bodies and improved lamellar bilayer formation at the stratum corneum-granular layer interface, demonstrating a capacity to restore the essential lipid "mortar" of the barrier [48]. It is noteworthy, however, that while this study highlights ceramide synthesis, systematic comparisons across studies are required to determine whether MSC-exos from different tissue sources (e.g., bone marrow versus umbilical cord) uniformly upregulate all critical lipid subclasses or exhibit distinct regulatory profiles.

Beyond lipids, MSC-exos enhance the expression of key structural proteins indispensable for barrier competence. In wound healing models, human adipose-derived MSC-exos (hADSC-Exo) upregulated the expression of FLG, loricrin, and aquaporin-3—proteins vital for terminal differentiation, cornified envelope assembly, and cutaneous hydration [49]. This pro-regenerative effect extends to modulating the dermal architecture. For instance, exosomes derived from human amniotic epithelial cells (hAECs-Exo) not only accelerated wound closure but also promoted more orderly collagen fiber alignment, suggesting a role in enhancing dermal resilience and minimizing scar formation [50]. A critical consideration is the variability in efficacy dependent on cellular origin. One in vitro comparative study found that exosomes from induced pluripotent stem cell-derived MSCs (iMSC-exos) exhibited a superior capacity to stimulate keratinocyte proliferation compared to their parent MSC-exos, potentially mediated through stronger activation of the ERK1/2 pathway [51]. This underscores that the cellular source of exosomes is a crucial variable influencing their bioactivity, a factor often insufficiently addressed in studies utilizing a single source.

The chemical acid mantle, maintained by factors such as filaggrin-derived urocanic acid and sebum, establishes a critical acidic microenvironment for optimal barrier function. Direct experimental evidence conclusively linking MSC-exos to the regulation of skin surface pH is currently lacking, representing a defined knowledge gap. Nevertheless, given their demonstrated role in modulating FLG metabolism and lipid synthesis [48, 49], it is plausible that MSC-exos contribute indirectly to stabilizing the chemical microenvironment necessary for acid mantle homeostasis. Future research should explicitly investigate this potential link by measuring pH dynamics and the expression of acid-producing enzymes following exosome treatment.

In summary, MSC-exos employ a coordinated strategy to repair the physical–chemical barrier axis, targeting lipid renewal, structural protein expression, and regenerative pathways. A critical appraisal of the literature confirms their therapeutic promise while simultaneously revealing a pressing need for more comparative, source-controlled studies. Rigorous head-to-head comparisons of exosomes derived from different sources and produced under standardized conditions are essential to identify the most potent and clinically viable candidates for barrier restoration therapies.

#### Restoring immune homeostasis and counteracting inflammation

Dysregulation of the skin's immune network is a central driver of chronic inflammatory diseases like AD and psoriasis. Within this complex pathological landscape, MSC-exos have emerged as sophisticated modulators, capable of targeting multiple dysregulated pathways. Their actions appear context-dependent, influenced by both disease pathophysiology and exosome source. In models of AD, ASC-exos effectively downregulate key Th2 cytokines such as IL-4, IL-13, and IL-31. Conversely, in psoriatic models, umbilical cord-derived exosomes predominantly attenuate the IL-17/IL-23 axis, highlighting their ability to engage distinct inflammatory circuits [55, 57]. This source-specific activity is further supported by cross-species evidence; for instance, canine adipose-derived MSC-exos ameliorated murine AD by inhibiting the JAK/STAT pathway and the pruritogenic receptor TRPA1 [58].

The immunomodulatory repertoire of MSC-exos extends beyond cytokine suppression to include the regulation of innate immune sentinels. Exosomes derived from tonsillar MSCs, for example, can directly inhibit TLR7-mediated mast cell activation, a pivotal event in the initiation of itch and inflammation [56]. Perhaps more profoundly, certain MSC-exos exhibit a capacity to promote immune tolerance. Studies indicate that systemic administration can foster the differentiation of regulatory T cells (Tregs), although this effect is contingent upon the presence of antigen-presenting cells, pointing to a refined, context-dependent mechanism for achieving antigen-specific tolerance [59]. A significant challenge for therapeutic translation lies in identifying the precise exosomal cargo responsible for this Treg expansion, as defining these active components is essential for standardizing efficacy.

The therapeutic profile of MSC-exos is further strengthened by their ancillary role in mitigating oxidative stress, a key amplifier of inflammatory tissue damage. By adaptively modulating the NRF2 signaling pathway, exosomes from sources like human umbilical cord MSCs bolster endogenous antioxidant defenses. This action helps protect keratinocytes and disrupt the vicious cycle linking oxidative stress and inflammation, a mechanism that also contributes to their observed protection against ultraviolet radiation-induced photoaging [60, 63].

Collectively, the evidence positions MSC-exos as multifaceted agents that can concurrently dampen pathogenic immune responses, regulate innate immunity, and potentially foster tolerogenic states, all while reducing associated oxidative damage. Advancing these findings will require a concerted effort to decode the specific immunomodulatory molecules within exosomes and to elucidate the pharmacokinetic principles that govern their targeted delivery to cutaneous immune cells, paving the way for their clinical development.

### Modulation of cutaneous sensory and homeostatic networks by MSC-exosomes: implications for the microbial, neural, and pigmentary interface

The skin's defensive capabilities extend beyond its well-characterized physical–chemical and immune barriers to include sophisticated interfacial systems—the microbial, neural, and pigmentary barriers. These systems function as dynamic signaling networks, engaged in continuous crosstalk to sense and adapt to both external and internal challenges. Dysfunction within one network frequently propagates imbalance to the others, contributing to the pathogenesis of complex dermatoses. Emerging preclinical evidence positions MSC-exos as promising multi-target agents capable of interacting with this interconnected axis to promote systemic homeostasis.

The cutaneous microbiome constitutes a critical chemical signaling interface, essential for educating the host immune system and modulating barrier function. Beneficial commensals, such as Staphylococcus epidermidis, reinforce barrier integrity by promoting keratinocyte differentiation and the production of AMPs. Current research suggests MSC-exos may support this interface primarily through host-directed immunomodulation. While direct evidence for exosome-specific effects is evolving, foundational studies on the MSCs secretome indicate it can stimulate keratinocyte AMP production via chemokines like CCL2 and disrupt pathogenic biofilms [53]. As a concentrated vesicular fraction, MSC-exos are postulated to deliver regulatory miRNAs and proteins that reprogram host keratinocyte and immune cell responses. This may indirectly suppress opportunistic pathogens like S. aureus and foster a microenvironment conducive to commensal stability, thereby leveraging the microbiome's role as a natural immune trainer.

Functioning as a vast neurosensory interface, the skin's network of nerve endings detects stimuli and releases neuropeptides that directly modulate vascular tone, immune cell activity, and keratinocyte function. Hyperactivity of this neuro-immune-cutaneous axis is a hallmark of conditions characterized by sensitivity and neurogenic inflammation. Preliminary clinical observations hint at the therapeutic potential of MSC-exos in this domain, with topical application reported to alleviate subjective symptoms such as stinging, itching, and burning in sensitive skin [54]. Mechanistically, this implies an ability to modulate the activity of sensory neurons and adjacent immune cells like mast cells, potentially attenuating hypersensitive neural signaling. However, the precise molecular targets—such as specific transient receptor potential (TRP) channels—require definitive elucidation to fully explain this dampening of neurogenic inflammation and its downstream effects.

As the optical signaling and defense interface, the pigment system, primarily through melanin, protects against UV radiation. Melanogenesis is intricately regulated by paracrine inflammatory and hormonal signals. Preclinical studies demonstrate that MSC-exos can provide targeted regulation at this interface. For instance, exosomes derived from human amniotic MSCs can inhibit UV-induced melanogenesis in vitro by delivering specific miRNAs (e.g., miR-181a-5p) that downregulate the master transcriptional regulator MITF [52]. Furthermore, evidence suggests they may enhance the autophagic clearance of melanosomes. This dual-action strategy—inhibiting synthesis while promoting degradation—points to a capacity to recalibrate melanocyte homeostasis in response to environmental stress.

In summary, the collective evidence underscores the potential of MSC-exos to engage multiple interfacial barriers—microbial, neural, and pigmentary—in a coordinated manner. Their action on one front, such as mitigating neurogenic inflammation, may favorably alter the microenvironment for another, like the microbial niche. This multimodal, network-level engagement theoretically addresses a fundamental pathology in complex skin disorders: the breakdown of integrated homeostasis across sensing and adaptive systems. Translating this potential into clinical reality necessitates a focused research agenda to decode the specific exosomal cargo responsible for these effects and to develop targeted delivery strategies that transform these versatile vesicles into precision therapies for dermatological care.

### Clinical applications of MSC-exos in dermatology

MSC-exos have emerged as a focus of significant interest in dermatology, supported by a growing number of clinical trials, with some applications already transitioning to clinical practice. These exosomes are being actively investigated, particularly in combination with advanced aesthetic technologies, for their potential in skin repair, anti-aging interventions, hair regeneration, and facial rejuvenation [64]. A randomized, active-controlled clinical study evaluated MSC-exos combined with transdermal techniques (NAFL) for melasma. Sixty patients were randomly assigned to four groups, including an active control (NAFL + saline). At six months, the combination therapy groups showed greater improvement in pigmentation (MASI score) and patient satisfaction than the control [65]. This study provides preliminary RCT-level evidence of potential efficacy; however, as a non-blinded trial with a modest sample size, confirmation from larger, double-blind RCTs is warranted.

In vitro evidence indicates that MSC-exos derived from human umbilical cord tissue promote fibroblast proliferation and type I collagen synthesis. Their demonstrated epidermal permeability in ex vivo human skin samples further supports their potential for anti-aging applications [66]. Although these preclinical findings strongly suggest utility in facial rejuvenation, direct clinical trial data remain scarce, and the safety and practicality of such applications necessitate further evaluation. Research in animal models suggests that the topical application of ASC-exos may enhance hair regeneration, potentially via upregulation of PDGF and VEGF [67]. This remains a preclinical finding, and its translational potential for human hair restoration therapy awaits future clinical validation.

Preclinical research has begun to address a primary challenge in MSC-exo therapy: their limited penetration into deeper skin layers. To overcome this, a sponge microneedle system derived from marine sponges (SHSs) has been developed. Studies in mouse and porcine skin models show that SHSs create microchannels, enhancing exosome penetration by nearly sixfold and significantly boosting their efficacy against UV-induced photoaging compared to exosomes alone [68]. While this strategy minimizes barrier disruption in animal models, its safety and efficacy in human skin remain to be evaluated in controlled clinical trials.

Research in animal models indicates that MSC-exos can orchestrate wound healing phases, favoring a regenerative outcome with reduced scarring [69]. These findings illuminate a potential cell-free strategy; however, its clinical utility for scar minimization in patients awaits confirmation in controlled trials.

In addition to their direct therapeutic use, MSC-exos can be engineered as advanced delivery vehicles by integrating with biomaterials. For instance, MSC-exos loaded with miR-155 inhibitors have been applied to diabetic wounds, where they significantly improved wound closure, epithelialization, and angiogenesis in both in vitro and animal models [70]. This strategy not only protects the nucleic acid payload from degradation but also enhances tissue-specific targeting and therapeutic efficiency, presenting a cost-effective treatment alternative. The capacity of MSC-exos to deliver functional miRNAs for skin barrier repair has been comprehensively reviewed [71], supporting their potential in managing barrier dysfunction-related diseases. Research in animal and cell models indicates that chitosan hydrogels can enhance the retention and efficacy of MSC-exos, leading to improved aging phenotypes in skin [72]. This work highlights a potential technical strategy; its translation into a clinical anti-aging therapy requires future investigation. MSC-exos also show promise as adjunctive therapies in dermatology. For example, in patients with AD who developed facial erythema as an adverse effect of dupilumab therapy, topical application of ASC-exos led to observable improvement. By modulating local inflammation and restoring barrier integrity, MSC-exos can mitigate such treatment-related side effects, thereby enhancing patient adherence and quality of life [73].

In conclusion, MSC-exos represent a versatile and promising therapeutic tool in dermatology, with applications ranging from skin barrier repair and anti-aging to hair regeneration and adjunctive therapy. Their integration with biomaterials and minimally invasive techniques opens new avenues for safe and effective treatments, addressing both aesthetic and medical dermatological needs.

## Challenges and directions

**Fig. 3:**
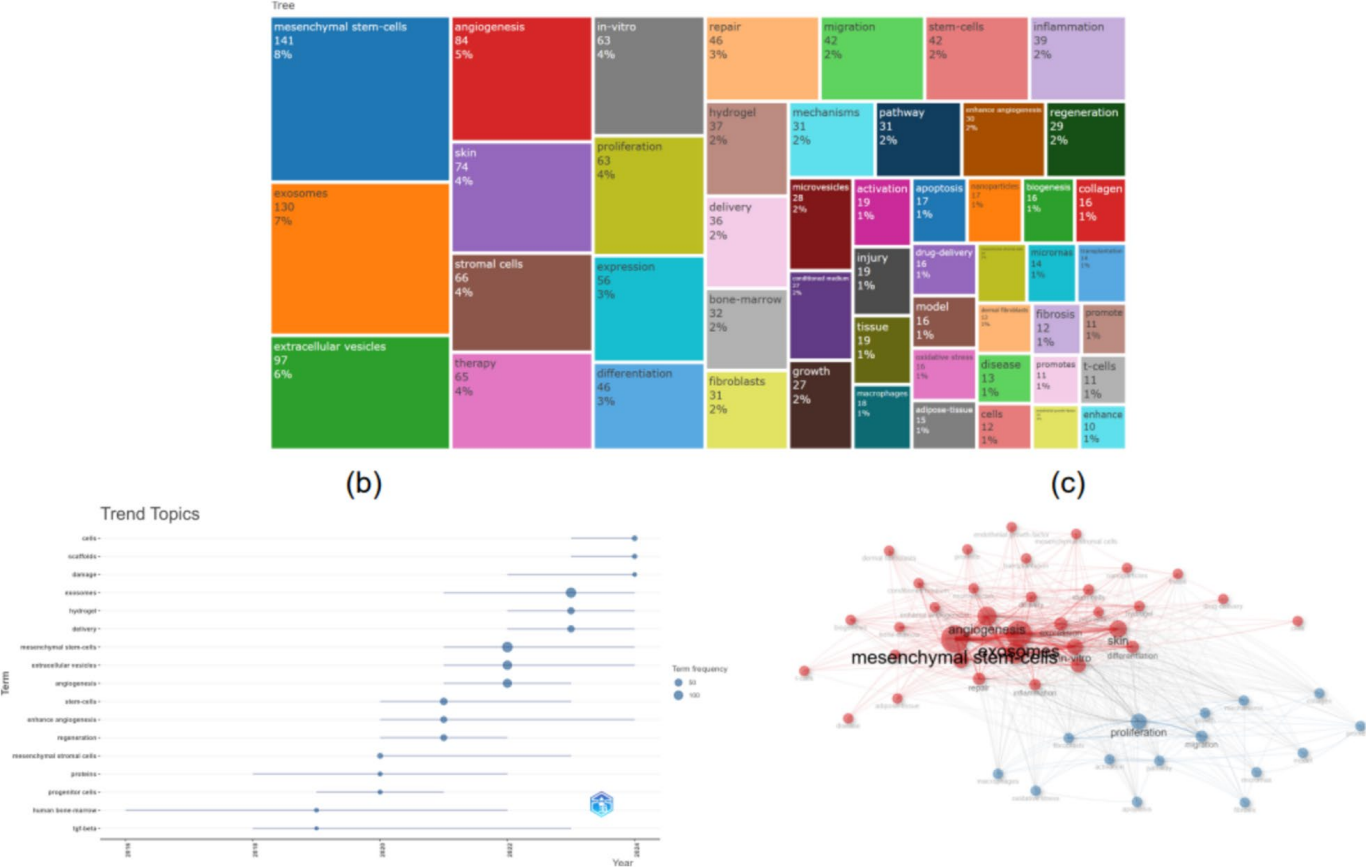
Bibliometric Analysis of Mesenchymal Stem Cell-Derived Exosomes in Skin-Related Research: Unveiling Future Research Hotspots and Potential Directions. **a** Keyword Tree Map: This visualization highlights the top 50 most frequently occurring keywords in the field. Beyond definition-related terms, high-frequency keywords include "angiogenesis," "therapy," "proliferation," "differentiation," "expression," "repair," "hydrogel," and "delivery." **b** Trend Analysis of Research Themes: The thematic evolution map reveals emerging research hotspots with significant potential in recent years, such as "hydrogel," "delivery," and "angiogenesis." **c** Keyword Co-Occurrence Network and Cluster Analysis: The analysis identifies two major research directions in the field. The blue cluster focuses on clinical applications, including "drug delivery," "angiogenesis," "tissue repair," "hydrogel," and "nanomaterials." The red cluster emphasizes fundamental research, covering topics such as "fibroblasts," "proliferation and apoptosis," "macrophages," "oxidative stress," and "migration."

To provide a quantitative overview of the research landscape, a bibliometric analysis was conducted. Publications from the Web of Science Core Collection and PubMed (2000–2023, English language) were analyzed using the bibliometrix R-package. Based on this analysis, the scientific challenges and application prospects of MSC-exos are discussed from multiple perspectives (Fig. 3).

### Clinical translation: integrating manufacturing standards with translational hurdles

The transition of MSC-exos from promising biologics to reliable clinical therapies necessitates overcoming interconnected challenges that span from production to patient application. Central to this effort is the establishment of robust manufacturing and quality control frameworks. This encompasses standardized protocols for donor screening, cell culture, and exosome isolation—a step where method choice (e.g., ultracentrifugation, size-exclusion chromatography) critically balances yield, purity, and bioactive integrity [74, 75]. Reproducible large-scale production under Good Manufacturing Practice (GMP) conditions remains a primary hurdle, as inconsistencies in these upstream processes lead to batch-to-batch heterogeneity [76].

This heterogeneity directly complicates the subsequent challenge of precise characterization and potency definition. Beyond essential release criteria based on identity (e.g., CD63/CD81) and physical attributes (e.g., concentration by nanoparticle tracking analysis), there is a pressing need to define robust potency assays. These assays must link specific molecular cargoes (e.g., key miRNAs or proteins) to biological function, moving beyond surrogate dose normalizers like total protein or particle count [59, 77].

Furthermore, pharmacokinetics and delivery present another layer of complexity. Understanding in vivo biodistribution and stability is essential. Strategies to enhance target-site retention, such as formulation within hydrogels or biomaterial scaffolds, are actively explored to improve therapeutic efficacy [78]. Finally, comprehensive safety and regulatory pathways must be solidified. While compiled preclinical and early clinical data are encouraging, with no serious adverse events directly attributed to MSC-exos reported to date [57, 69], comprehensive long-term safety profiles and globally harmonized regulatory guidelines for exosomes as therapeutic agents are still under development [76].

In conclusion, advancing MSC-exos into mainstream dermatological therapy requires a holistic approach that seamlessly integrates standardized production, rigorous characterization, and tailored delivery, all within a maturing safety and regulatory landscape. Addressing these interdependent challenges through interdisciplinary collaboration is paramount for harnessing their full therapeutic potential.

### Addressing challenges in mesenchymal stem cell sources

Current research on MSCs predominantly utilizes sources such as adipose tissue, bone marrow, and umbilical cord. However, these origins are often accompanied by ethical considerations and difficulties in ensuring cellular homogeneity. To overcome these constraints, alternative MSC sources with potent immunomodulatory properties and fewer ethical complications are being explored.Dermal mesenchymal stem cells (DMSCs), while less investigated in dermatology compared to their bone marrow, adipose, or umbilical cord counterparts, have demonstrated multipotent differentiation and self-renewal capacity in vitro, particularly toward neural lineages [79]. Their easy accessibility and widespread distribution make them attractive; however, evidence supporting their immunosuppressive functions and full compliance with internationally established MSC criteria remains limited, highlighting a promising yet underexplored area for future study [73].Induced pluripotent stem cells (iPSCs), reprogrammed from somatic cells, present a viable alternative by generating highly homogeneous MSC populations (iMSCs) that adhere to international standards and bypass ethical concerns. Such iMSCs could provide a scalable and consistent source for MSC-exos. Nevertheless, several challenges—including comprehensive safety assessment, exosome quality control, and the complexity of isolation protocols—must be addressed before clinical translation can be realized [80].

### Preconditioning MSCs with specific factors for precision medicine

The biological functions of MSCs and their exosomes can be significantly modulated by their extracellular milieu—a principle leveraged through preconditioning strategies. For instance, exposure to granules from mast cells (MCs), which are key sentinel cells in innate immunity and allergy, enhances the proliferative and immunomodulatory capacity of human umbilical cord blood-derived MSCs (hUCB-MSCs). When subcutaneously injected into AD mouse models, these preconditioned MSCs inhibited MC degranulation and proliferation, reduced serum IgE levels, suppressed B-cell maturation, and markedly alleviated AD lesions [81]. Similarly, preconditioning MSCs with interferon-gamma (IFN-γ) augments their immunoregulatory potential. Exosomes derived from IFN-γ-preconditioned MSCs demonstrated superior efficacy in ameliorating AD symptoms in vivo, achieved through the suppression of key inflammatory cytokines (IL-4, IL-31, IL-13) and inhibition of Th2 cell activation [82]. These findings underscore how targeted preconditioning can optimize the therapeutic profile of MSC-exos for specific inflammatory conditions.

In diabetic wound healing, preconditioning MSC-exos with pioglitazone, a drug used for diabetes management, promotes vascular endothelial cell viability and proliferation under high-glucose conditions, accelerating wound healing and angiogenesis in diabetic rat models [83]. These findings highlight the potential of preconditioning MSCs with specific factors to enhance their therapeutic efficacy and achieve precision medicine.

### Limitations and future directions in MSC-exo-mediated skin barrier repair

While substantial evidence supports the role of MSC-exos in repairing key skin barriers (physical, immune), their effects on other dimensions—such as the chemical, neuro-immune, and pigment barriers—remain less defined and warrant targeted investigation. For instance, despite promising results in hair regeneration, the potential of MSC-exos to regulate sebaceous gland function and sebum production is unexplored and represents a novel avenue for managing sebum-related disorders.

Future progress hinges on elucidating the precise molecular mechanisms and bioactive components within MSC-exos. Deeper profiling of their cargo (proteins, miRNAs) may uncover new therapeutic agents for a broad spectrum of cutaneous diseases.

For clinical translation, a rational choice of administration route is critical, as it directly dictates biodistribution and therapeutic potential. Based on existing evidence, these routes can be broadly ranked in expected tissue exposure and persistence: topical (epidermal, limited systemic exposure) < subcutaneous (dermal/local, sustained reservoir) < intravenous (systemic, potential for targeted homing). Consequently, the comparative safety, efficacy, and practicality of different routes (transdermal, subcutaneous, intravenous) must be systematically evaluated in disease-specific contexts.

Owing to their innate capabilities in immunomodulation, barrier restoration, and drug delivery, MSC-exos hold significant promise not only for therapeutic skin repair but also for applications in medical aesthetics and advanced cosmeceuticals. Realizing this full potential will require bridging the current mechanistic gaps with rigorous, application-driven research.

### Combining MSC-exos with other therapeutic modalities

MSC-exos can act not only as standalone therapeutic agents for skin barrier repair and disease treatment but can also be integrated with exosomes from other cellular, plant, or animal sources to engineer multifunctional hybrid nanovesicles for personalized therapeutic platforms. For example, hybrid vesicles combining MSC-exos with grapefruit-derived exosomes—known for their inherent anti‑inflammatory and antioxidant properties—have demonstrated enhanced therapeutic efficacy in models of psoriasis and AD [84]. Such multifunctional systems extend beyond local tissue repair to confer systemic immunomodulatory effects.

The growing interest in oral exosome therapy for various diseases [85, 86] further supports the potential of designing targeted hybrid nanovesicles by fusing MSC-exos with exosomes from biological sources that exhibit natural tropism to the skin. However, the potential risks and biological challenges arising from cross-species RNA, miRNA, and other molecular interactions must be thoroughly evaluated before clinical translation.

## Conclusions

The skin barrier is a cornerstone in the pathogenesis and clinical management of numerous dermatological diseases. Yet, its complex, multi-layered structure and robust intercellular integrity have posed significant challenges to the development of effective therapeutics. In recent years, exosomes—particularly MSC-exos—have emerged as a transformative, cell-free therapeutic paradigm. Their appeal lies in a combination of biosafety, potent efficacy, and versatility as natural delivery vehicles.

MSC-exos exhibit a remarkable capacity to modulate immune responses and promote tissue repair. Their multifaceted actions enable them to restore homeostasis in dysregulated skin environments, making them uniquely suited to address the composite nature of skin barrier dysfunction. As this review has detailed, MSC-exos from diverse sources show promising efficacy in restoring the physical, immune, microbial, and antioxidant barriers of the skin.

Looking forward, realizing the full therapeutic potential of MSC-exos will require continued efforts to elucidate their precise molecular mechanisms, standardize isolation and characterization protocols, and evaluate optimal delivery strategies. Future research should also explore innovative approaches, such as bioengineering exosomes or developing hybrid nanovesicles, to enhance targeting and functionality. By integrating these advances, MSC-exo-based therapies are poised to offer novel, precise, and effective solutions for skin barrier repair and the treatment of a broad spectrum of dermatological conditions.

## Data Availability

All the data and materials supporting the conclusions were included in the main paper.

## Abbreviations

MSC: Mesenchymal stem cell
BMSC: Bone marrow-derived mesenchymal stem cell
ASC: Adipose-derived mesenchymal stem cell
UCMSC: Umbilical cord-derived mesenchymal stem cell
MSC-exo: Mesenchymal stem cell-derived exosome
AD: Atopic dermatitis
TEWL: Transepidermal water loss value
FLG: Filament polyprotein
TSLP: Thymic stromal lymphopoietin
AMP: Antimicrobial peptide
TNF: Tumor necrosis factor
MC: Mast cell
UV: Ultraviolet
LB: Lamellar body
SG: Stratum granulosum
TJ: Tight junction
hASC: Human adipose-derived mesenchymal stem cell
hASC-exo: Human adipose mesenchymal stem cell-derived exosome
SC: Stratum corneum
AHR: Aryl hydrocarbon receptor
MSC-CM: Mesenchymal stem cell extracellular matrix
SP: Serine protease
NAFL: Nonablative fractional resurfacing
PBASM: Peninsula Blue Aurora Shumin Master
iPSC: Induced pluripotent stem cell
iMSC: Induced pluripotent mesenchymal stem cell
